# Fish and Meat

**Published:** 1870-11

**Authors:** 


### FISH AND MEAT.

It has been said that fish food is adapted to thinkers, because, having more phosphorus, it is peculiarly adapted to feed the brain ; it is not as nutritious as poultry or beef, but it is quite as healthful.

It is not wise to eat by rules made in the chemical laboratory, or in the study of the philosopher. Almighty wisdom has placed the preservation and development of the species as he has that of the sex, beyond human ken, and has implanted within us certain uncontrollable instincts, which prompt us to live, and move, and sustain our beings with a greater wisdom than could be hoped from mortal agencies, comprehended in the single dictation, "Eat what you feel like"—that is, partake in moderation of what is most palatable to you ; but if, in rare cases, it is found that what you are most fond of is followed by disagreeable results, gracefully yield to nature, avoid it for a while at least, and you will find that what does not agree with you to-day, may be actually beneficial next month or next year.

